# *Mycobacterium avium* complex pulmonary disease patients with limited treatment options

**DOI:** 10.1183/23120541.00610-2023

**Published:** 2024-01-15

**Authors:** Stefano Aliberti, Francesco Blasi, Pierre-Régis Burgel, Andrea Calcagno, Andreas Fløe, Dorothy Grogono, Apostolos Papavasileiou, Eva Polverino, Concepción Prados, Gernot Rohde, Helmut J.F. Salzer, Adrián Sánchez-Montalvá, Michal Shteinberg, Eva Van Braeckel, Jakko van Ingen, Nicolas Veziris, Dirk Wagner, Michael R. Loebinger

**Affiliations:** 1 Department of Biomedical Sciences, Humanitas University, Milan, Italy; 2 IRCCS Humanitas Research Hospital, Respiratory Unit, Milan, Italy; 3 Department of Pathophysiology and Transplantation, Università degli Studi di Milano, Milan, Italy; 4 Internal Medicine Department, Respiratory Unit and Cystic Fibrosis Center, Fondazione IRCCS Cà Granda Ospedale Maggiore Policlinico Milano, Milan, Italy; 5 Respiratory Medicine, Université Paris Cité, Inserm U1016, Institut Cochin, Paris, France; 6 Cochin Hospital, Department of Respiratory Medicine, Publique Hôpitaux de Paris, Paris, France; 7 Department of Medical Sciences, Infectious Diseases, University of Turin, Turin, Italy; 8 Department of Respiratory Diseases, Aarhus University Hospital, Aarhus, Denmark; 9 Cambridge Centre of Lung Infection, Royal Papworth Hospital NHS Foundation Trust, Cambridge, UK; 10 “Sotiria” General Hospital of Chest Diseases, Athens, Greece; 11 Respiratory Medicine, Adult Bronchiectasis and Cystic Fibrosis, University Hospital Vall D'Hebron, VHIR, CIBERES, Barcelona, Spain; 12 Pulmonology, Cystic fibrosis, Bronchiectasis, Bronquial Infections, La Paz University Hospital, Madrid, Spain; 13 Goethe University Frankfurt, University Hospital, Medical Clinic 1, Respiratory Medicine and Allergology, Frankfurt am Main, Germany; 14 Division of Infectious Diseases and Tropical Medicine, Department of Internal Medicine 4 - Pneumology, Kepler University Hospital, Linz, Austria; 15 Medical Faculty, Johannes Kepler University Linz, Linz, Austria; 16 Ignaz Semmelweis Institute, Interuniversity Institute for Infection Research, Vienna, Austria; 17 International Health Unit Vall d'Hebron-Drassanes, Infectious Diseases Department, Vall d'Hebron University Hospital, PROSICS, Universitat Autónoma de Barcelona, Barcelona, Spain; 18 Centro de Investigación Biomédica en Red de Enfermedades Infecciosas (CIBERINFEC), Instituto de Salud Carlos III, Madrid, Spain; 19 Micobateria Infection Study Group (GEIM) from Spanish Society of Infectious Diseases and Clinical Microbiology (SEIMC), Madrid, Spain; 20 Carmel Medical Center and the Technion-Israel Institute of Technology, B. Rappaport Faculty of Medicine, Haifa, Israel; 21 Department of Respiratory Medicine, Ghent University Hospital, Ghent, Belgium; 22 Respiratory Infection and Defense Lab (RIDL), Department of Internal Medicine and Paediatrics, Ghent University, Ghent, Belgium; 23 Clinical Microbiologist, Medical Microbiology, Radboud University Medical Center, Nijmegen, The Netherlands; 24 Sorbonne Université, Centre d'Immunologie et des Maladies Infectieuses (Cimi-Paris), UMR 1135, Department of Bacteriology, Saint-Antoine Hospital, APHP, Sorbonne-Université, Centre National de Référence des Mycobactéries, Paris, France; 25 Department of Internal Medicine II, Division of Infectious Diseases, Medical Center – University of Freiburg, Faculty of Medicine, University of Freiburg, Freiburg, Germany; 26 Host Defence Unit, Royal Brompton Hospital, and NHLI, Imperial College, London, United Kingdom

## Abstract

Over recent decades, the prevalence and incidence of nontuberculous mycobacterial pulmonary disease (NTM-PD) have increased worldwide, with *Mycobacterium avium* complex being the most common causative agents [1–5]. The 2020 American Thoracic Society/European Respiratory Society/European Society of Clinical Microbiology and Infectious Diseases/Infectious Diseases Society of America (ATS/ERS/ESCMID/IDSA) guidelines recommended a treatment regimen with at least three drugs, including a macrolide, in patients with a nodular–bronchiectatic, macrolide-susceptible *M. avium* complex pulmonary disease (MAC-PD) [6].

## Tweetable abstract

**How to identify MAC-PD patients with limited treatment options: an expert consensus** https://bit.ly/3QwLQ8T

*To the Editor:*

Over recent decades, the prevalence and incidence of nontuberculous mycobacterial pulmonary disease (NTM-PD) have increased worldwide, with *Mycobacterium avium* complex being the most common causative agents [1–5]. The 2020 American Thoracic Society/European Respiratory Society/European Society of Clinical Microbiology and Infectious Diseases/Infectious Diseases Society of America (ATS/ERS/ESCMID/IDSA) guidelines recommended a treatment regimen with at least three drugs, including a macrolide, in patients with a nodular–bronchiectatic, macrolide-susceptible *M. avium* complex pulmonary disease (MAC-PD) [6]. For patients with cavitary or advanced/severe bronchiectatic disease, guidelines suggest the addition of parenteral amikacin into the initial regimen [6]. Once treatment is initiated, up to 40% of NTM-PD patients might experience an unsuccessful outcome [7, 8]. For the first time, the 2020 ATS/ERS/ESCMID/IDSA guidelines identified patients with a “refractory” MAC-PD as those with a positive sputum culture after 6 months of guideline-based therapy (GBT) [6]. In refractory MAC-PD, a recommendation was made in the guidelines to add amikacin liposome inhalation suspension (ALIS) to the treatment regimen [6]. This recommendation was based on the CONVERT study, demonstrating an improved culture conversion rate in refractory MAC-PD [6, 9]. Based on those results, the US Food and Drug Administration (FDA) approved ARIKAYCE, a proprietary ALIS formulation by Insmed, Inc. (Bridgewater, NJ, USA), for “treatment-refractory MAC lung disease” [10]. The European Medicines Agency (EMA) went further, licensing ARIKAYCE to treat NTM lung infections “caused by MAC in adults with limited treatment options who do not have cystic fibrosis” [11]. However, the term “limited treatment options” (LTOs) might sound ambiguous, and no clinical study, expert consensus or guidelines exist to clarify the meaning of limited treatment options. The experts convened on such ambiguity in their preliminary discussion and decided to possibly contribute to shedding light on the issue term. With regards to the reimbursement of ARIKAYCE, the product is generally covered by health insurers in the USA and is fully reimbursed by the National Health Insurance of Japan. In Europe, the product is fully reimbursed in the UK, France, Ireland, Belgium, the Netherlands and Finland. In Germany, Denmark and Greece, it is generally funded for individual patients.

To reach a consensus on common clinical scenarios of LTOs for MAC-PD patients and to identify which MAC-PD patients with LTOs could benefit from ALIS, a panel of experts in respiratory and infectious diseases, including tuberculosis, from continental Europe, the UK and Israel convened in Milan, Italy, on 22–23 February 2023. The focus was on the ambiguous EMA definition, leading to the decision to include only European and Israeli experts; a perhaps limiting decision. However, the consensus outcomes could also be of some help to NTM specialists outside Europe.

A thorough search of the published high-level literature on the management of NTM-PD and MAC-PD patients, and its authors, was the basis for identifying and selecting the roster of 18 candidate European and Israeli experts. All 18 invited experts, including A. Fløe, C. Prados, A. Sánchez-Montalvá and J. van Ingen, contributed to online discussions before the face-to-face Milan meeting; 14 of the identified experts also participated in the face-to-face event: S. Aliberti, F. Blasi, P-R. Burgel, A. Calcagno, D. Grogono, M.R. Loebinger, A. Papavasileiou, E. Poliverino, G. Rohde, H.J.F. Salzer, M. Shteinberg, E. Van Braeckel, N. Veziris and D. Wagner. Similarly, a thorough literature search helped to identify all the available evidence covering MAC-PD patients with LTOs. The selected manuscripts were shared among the reviewers and carefully reviewed to contextualise the topic. An online collection of real-life MAC-PD clinical situations personally confronted by the panel experts followed, with the development of a draft set of real-life cases of possible MAC-PD patients with LTOs from those real-life cases. Before the Consensus Conference, the panel experts voted blindly online to decide if they considered the cases as LTO situations with the following voting options: “Yes, an LTO example”, “No, not an LTO example” and “Not enough information to decide”. Draft examples without full expert endorsement were either modified or discarded. During the face-to-face meeting in Milan, the experts discussed and altered the iconic illustrations and their descriptors (“statements”) to reach a consensus about their relevance as realistic clinical possibilities. As a final step, the panel members voted on each statement using a modified Delphi method. The double goals of the voting process, with scores variable from 0 to 10, were to confirm whether each iconic statement might qualify as an actual MAC-PD LTO and whether ALIS might have a role in the real-life management of such situations.

All experts considered refractory MAC-PD (according to the 2020 ATS/ERS/ESCMID/IDSA guidelines definition) as an LTO situation (100% agreement), including both smear-negative and smear-positive disease, and to include ALIS in their treatment strategy. Some experts would also consider using intravenous amikacin in some refractory conditions with evidence of continuing severe disease. In smear-positive, nodular–bronchiectatic, refractory MAC-PD, two experts would start *i.v.* amikacin before shifting to ALIS. In cavitary refractory MAC-PD, one expert would start *i.v.* amikacin before turning to ALIS. The FDA license and the clinical guidelines based on the evidence provided by the CONVERT study cover refractory MAC-PD. However, given the wording of the EMA licensing, the expert panel identified seven potential LTOs for MAC-PD patients (table 1). The panel also debated whether the now available ALIS formulation could be speculatively used with benefit in each of these situations while unanimously acknowledging that current evidence is for refractory MAC-PD only. At any rate, all the described speculative scenarios cannot and should not be equated to the EMA's definition of LTOs. Confirmatory validation through targeted clinical trials would still be a must.

**TABLE 1:** Limited treatment options for patients with *Mycobacterium avium* complex pulmonary disease (MAC-PD) in the panel's opinion

**Scenario**	**Is this a limited treatment option situation?**	
	**Yes**	**No**
**Refractory MAC-PD according to current official ATS/ERS/ESCMID/IDSA clinical practice guideline definition [** **6** **]**	14 (100%)	0
**Macrolide-resistant strain**		
Newly diagnosed, noncavitary MAC-PD due to a macrolide-resistant, amikacin-susceptible (≤64 µg·mL^−1^) strain	14 (100%)	0
Newly diagnosed, noncavitary MAC-PD due to a macrolide-resistant, amikacin-intermediate strain	14 (100%)	0
Newly diagnosed, noncavitary MAC-PD due to a macrolide-resistant, amikacin-resistant (>64 and <128 µg·mL^−1^) strain	14 (100%)	0
**Inability to take or tolerate drugs**		
Macrolides, for whatever reason	14 (100%)	0
Ethambutol, for whatever reason	14 (100%)	0
Rifamycins, for whatever reason	3 (21%)	11 (79%)
Intravenous amikacin when indicated (*e.g.* cavitary disease) for whatever reason	14 (100%)	0
**Relapse^#^**	14 (100%)	0
**Re-infection^¶^**	0	14 (100%)

ATS/ERS/ESCMID/IDSA: American Thoracic Society/European Respiratory Society/European Society of Clinical Microbiology and Infectious Diseases/Infectious Diseases Society of America. ^#^: according to the NTM-NET definition [12], occurring in a patient who underwent guideline-based therapy, assuming it is not reinfection; ^¶^: according to the NTM-NET definition [12], after stopping treatment.

All 14 voting members of the panel agreed that newly diagnosed, noncavitary MAC-PD caused by a macrolide-resistant strain represents an LTO, regardless of whether the strain is amikacin-susceptible or amikacin-intermediate [13]. All experts agreed that some patients might derive potential benefits from ALIS in this group, although with the understanding that there is no direct evidence for the benefit of ALIS in this specific setting. The experts also recognised newly diagnosed MAC-PD caused by a macrolide-resistant and *i.v.* amikacin-resistant strain as an LTO situation. The 2018 Clinical and Laboratory Standards Institute M24-A3 and M62 guidelines and the ATS/ERS NTM guideline laboratory section support the use of ALIS with *i.v.* amikacin-resistant strains if the amikacin minimum inhibitory concentration (MIC) is 64 µg·mL^−1^ associated with *i.v.* amikacin resistance and ALIS susceptibility, due to high local concentrations and potentially improved intracellular penetration of the liposomal formulation [6, 14, 15]. All experts agreed that the proprietary ALIS formulation should not be an option if amikacin MICs are ≥128 µg·mL^−1^. Seven experts (50%) acknowledged the role of ALIS in the macrolide-resistant, amikacin-resistant (>64 and <128 µg·mL^−1^) strain condition.

Regarding MAC-PD patients intolerant of a GBT regimen for any reason, the outcomes of discussions were variable. If feasible, switching within the macrolide class should consistently preserve a macrolide-based regimen. Premature discontinuation of the macrolide because of an inability to take the drug was considered an LTO situation by all the experts, who also unanimously (100%) identified a potential role of ALIS in this case. Some experts also argue about initiating *i.v.* amikacin according to the patient's preferences or needs. The inability to take ethambutol was also recognised as an LTO situation, with most experts not identifying any role for the ALIS formulation (11 out of 14 voting panel members, 79%). An oral drug, such as clofazimine, seemed advisable in cases of intolerance to the “companion” drug ethambutol [6]. Intolerance of rifamycins was regarded as an LTO situation by only three experts out of 14 (21%), with emerging data and ongoing clinical trials (www.clinicaltrials.gov identifier numbers NCT03672630 and NCTO4677569) investigating their concrete benefit in MAC-PD patients [16]. In these cases, the advice was to try switching within the rifamycin class or considering clofazimine as a third drug with no role for ALIS. Finally, when suggested by guidelines (*e.g.* cavitary disease), the inability to use *i.v.* amikacin was also considered an LTO situation by all the experts who unanimously (100%) agreed that ALIS administration could be an option in this case.

Relapses in a GBT-treated patient were the final situation discussed, unanimously (100%) recognised as LTO conditions with a role for the proprietary ALIS formulation for all experts. Provided there was adequate compliance to GBT, drug susceptibility testing and the frequency of microbiology work-up are essential when deciding on treatment. It is crucial to try differentiating relapse from re-infection by genome sequencing, with relapse considered similar to a refractory situation. All experts agreed that re-infections are not an LTO situation.

This expert panel discussion and consensus might help physicians interpret EMA documents referring to LTO in MAC-PD patients. However, it is essential to understand that the value of the document is in discussing which conditions may relate to MAC-PD patients with LTO and in which of these the panel considers that there could be some potential benefit of using ALIS with the knowledge that, at present, evidence and guidelines on the use of ALIS relate to refractory MAC-PD.
